# *Lactiplantibacillus plantarum* KABP051: Stability in Fruit Juices and Production of Bioactive Compounds During Their Fermentation

**DOI:** 10.3390/foods13233851

**Published:** 2024-11-28

**Authors:** Francesca Rizzi, Bibiana Juan, Jordi Espadaler-Mazo, Marta Capellas, Pol Huedo

**Affiliations:** 1 Centre d’Innovació, Recerca i Transferència en Tecnologia dels Aliments (CIRTTA), XIA, TECNIO, Departament de Ciència Animal i dels Aliments, Facultat de Veterinària, Universitat Autònoma de Barcelona (Cerdanyola del Vallès), 08193 Barcelona, Spain; rizzi@ab-biotics.com (F.R.); bibiana.juan@uab.cat (B.J.); marta.capellas@uab.cat (M.C.); 2 R&D Department, AB-Biotics S.A. (Part of Kaneka Corporation), 08174 Barcelona, Spain; espadaler@ab-biotics.com; 3 Basic Sciences Department, Universitat Internacional de Catalunya, 08195 Barcelona, Spain

**Keywords:** probiotics, fermentation, functional foods, bioactive compounds, fruit juices

## Abstract

The lactic fermentation of fruit and vegetable juices by well-characterised probiotics remains relatively underexplored. We have investigated the stability and impact of *Lactiplantibacillus plantarum* KABP051 fermentation on orange, apple, and peach juices by microbiological, physicochemical, and sensory evaluation means. For each fruit juice, three different samples were analysed: original fruit juice without probiotic as blank (B), fruit juice inoculated with 10^7^ CFU/mL of probiotic without fermentation (P), and fruit juice inoculated with 10^7^ CFU/mL of probiotic and fermented at 37 °C for 24 h (PF). P samples displayed good stability throughout the study, and PF samples showed an initial increase in CFUs accompanied by a change in pH, confirming the ability of the probiotic to ferment these juices. After 60 days of refrigeration, PF samples contained >10^7^ CFU/mL. Total phenolic content and antioxidant capacity were equivalent in F, P, and PF. Remarkably, deep metabolomic analyses confirmed malolactic fermentation and revealed the production of several bioactive compounds including the antimicrobial substance phenyllactic acid, the immunomodulatory and anti-fatigue amino acid N-acetyl glutamine, the vitamin B3 form nicotinic acid, the monoterpene (−)-β-pinene, and the neurotransmitter acetylcholine, among others, during probiotic fermentation. Finally, a hedonic analysis involving 51 participants showed that probiotic fermented orange juice is well accepted by panellists, with scores comparable to those of the control juice. Overall, we here show that fruit juices are excellent carriers for the delivery of the probiotic *L. plantarum* KABP051 and its non-alcoholic fermentation can result in tasty functional fruit juices enriched with health-promoting compounds.

## 1. Introduction

In recent years, and especially after the pandemic period, consumers have gained awareness about the importance of preventive health. In this context, gut microbiota is gaining momentum, with increasing evidence on the importance of maintaining its balance for human health [1,2]. It is known that gut microbiota composition not only influences the digestive system but also has an impact on distant organs and systems by the different gut–organ axes, including the so-called gut–brain, gut–liver, gut–lung, gut–muscle and gut–skin axes [3,4,5,6,7,8]. Different probiotics have shown efficacy in the modulation of these axes, exerting beneficial effects beyond the digestive tract [9].

The term “functional food”, apparently coined in Japan in the 1980s–90s, defines a food that, beyond basic dietary benefits, exerts additional health effects in the body [10]. Probiotics, in line with the latter term, are defined as “live microorganisms that, when administered in adequate amounts, confer a health benefit on the host” [11]. Recently, the International Scientific Association for Probiotics and Prebiotics (ISAPP) has defined the terms “fermented food” and “probiotic fermented food” [12]. According to ISAPP, “fermented foods” are “foods made through desired microbial growth and enzymatic conversions of food components”, and “probiotic fermented food” are “food fermented by or containing probiotic(s) with strain-specific evidence”. As per definition, the number of live probiotic cells must be sufficient to exert the beneficial effects validated in preclinical and clinical studies throughout the shelf life of the product [12,13].

Fermented foods are the oldest and most traditional version of functional foods and have been part of the human diet for millennia [14,15,16]. Several bioactive metabolites have been identified as a result of bacterial protein, lipid, and carbohydrate metabolism during the fermentation process. The production of vitamins and antioxidants as well as an increase in the bioavailability of minerals has been reported for many lactic acid bacteria species [17]. A wide assortment of biologically active peptides of bacterial origin has also been described in fermented products displaying antioxidant, anti-microbial, anti-fungal, anti-inflammatory, anti-diabetic, and anti-atherosclerotic activity, among others [18]. Thus, fermented probiotic products, which contain active bacteria and biomolecules, exert health-promoting effects by modulating the intricated network that involves probiotic-, biomolecules- gut microbiota-, and host interactions.

Food and beverage matrices are a combination of countless sets of physicochemical parameters and conditions (pH, water activity, temperature, etc.) that may affect probiotic viability. Moreover, each probiotic strain has different traits and growth and survival needs [11]. Therefore, every probiotic strain should be individually investigated and selected according to the characteristics of the food and the microbe.

Historically, most marketed probiotic fermented foods have been of dairy origin, limiting their utilisation by consumers following strict vegetarian or vegan diets, as well as those with food intolerances. Therefore, the addition of probiotics to fruit juices, a plant-based matrix, could not only increase the nutritional value of the product but also benefit a wider population [19].

Fruits are naturally rich in minerals, vitamins, dietary fibers, antioxidants, and many other beneficial nutrients that make them essential components of a healthy and balanced diet. Due to new healthy trends, sales of fruit and vegetable juices have increased in recent years [20].

*Lactiplantibacillus plantarum* is frequently used as a starter or adjunct cultures in fermentation of raw materials from plant and animal origin because of its environmental resilience and metabolic versatility [21]. *L. plantarum* strains display a high level of genome diversity, yet their shared core genome reveals a remarkably nomadic lifestyle, ranging from fruits and vegetables to the intestinal tract of herbivores and omnivores, stools, urine, or milk [22]. *L. plantarum* KABP051 is one such nomadic strain, independently isolated both from human faeces and cow’s milk, which has shown a beneficial effect in various clinical trials [23,24,25,26] and been subjected to extensive characterisation. For these reasons, we selected this strain to investigate its behaviour in natural fruit juices.

The aim of the present study was to assess the stability (viability) of the probiotic *L. plantarum* KABP051 in orange, apple, and peach juices during two months of refrigerated storage, and to investigate the impact of its presence and fermentation on the physicochemical, nutritional, and organoleptic characteristics of the juices by microbiological, metabolomic, and sensory evaluation approaches.

## 2. Materials and Methods

### 2.1. Materials

#### 2.1.1. Bacterial Strains

A freeze-dried culture of the probiotic lactic acid bacteria *Lactiplantibacillus plantarum* KABP051 (CECT7481, DR7^TM^) was supplied by AB-Biotics SA (KANEKA Corp., Barcelona, Spain) and stored at −20 °C in a sealed aluminium bag until use.

#### 2.1.2. Fruit Juices

Three different commercial UHT 100% fruit juices were purchased in local supermarkets in Barcelona, Spain, from local brands: orange (“Orange juice without pulp”, Hacendado, Barcelona, Spain), apple (“Organic apple juice”, Bonpreu, Barcelona, Spain), and peach (“Organic peach juice”, Bonpreu, Barcelona, Spain).

### 2.2. Methods

#### 2.2.1. Inoculation and Fermentation of Fruit Juices

To obtain an initial probiotic concentration of 10^7^ CFU/mL (corresponding to 10^9^ CFU/serving in 240 mL), all juices were inoculated with the freeze-dried *L. plantarum* strain KABP051 under aseptic conditions. For each fruit juice, three different samples were prepared: original fruit juice without probiotic as blank (B), fruit juice with 10^7^ CFU/mL of probiotic without fermentation (P), and fruit juice inoculated with 10^7^ CFU/mL and fermented at 37 °C for 24 h (PF). Samples were stored at 4 °C for 60 days (Figure S1).

#### 2.2.2. Stability Analyses

Microbiological and physicochemical changes in the fruit juices were determined at days 0 (before and after inoculation), 1, 7, 30, and 60 to assess product quality and probiotic stability as follows:

##### Physicochemical Analyses

B, P, and PF juices were tempered to 20 °C before physicochemical analyses. pH was measured using a pH-Meter (Basic 20 Crison Instruments, S.A., Barcelona, Spain), and total soluble solids (TSS) were analysed using an optical refractometer (Analogue hand-held refractometer PREF-B32-001, Labbox Labware, S.L., Barcelona, Spain), and the results were reported as ºBrix [27].

##### Bacterial Viable Count

CFU enumeration was determined by the standard plate count method in triplicate. Serial dilutions of probiotic juices were prepared using a peptone buffer, and 100 μL of the appropriate dilutions were seeded onto de Man, Rogosa, and Sharpe (MRS) agar and incubated anaerobically at 37 °C for 48 h. To discard bacterial contamination, samples were analysed before probiotic inoculation and at the end of the study. The presence of coliforms, mesophilic aerobic bacteria, psychrotrophic bacteria, and yeasts/moulds was investigated by seeding samples onto Chromogenic Coliform Agar, Plate Count Agar, and Rose-Bengal Chloramphenicol Agar. These plates were incubated 24 h at 37 °C, 72 h at 30 °C, 7 days at 7 °C, and 7 days at 30 °C, respectively.

#### 2.2.3. Determination of Total Phenolic Content

Total phenolic content in B, P, and PF samples was determined by using an adaptation of the Folin–Ciocalteu (F-C) assay [28]. In brief, 1.97 mL of Milli-Q water and 175 µL of Folin & Ciocalteu’s phenol reagent were added to 100 μL of the appropriate filtered and diluted sample, standard or blank. Milli-Q water, instead of juice, was used for the control. After 8 min of incubation at room temperature, 375 µL of 20% Na_2_CO_3_ solution were added, and the mixture was incubated for 30 min in complete darkness at 40 °C. The final mixture absorbance was measured using a spectrophotometer at 765 nm.

A gallic acid standard calibration curve (0–500 mg/L) was used for the quantification of total phenolic compounds, and the results were expressed as gallic acid equivalent (GAE) using units of mg/L.

#### 2.2.4. Determination of DPPH Radical Scavenging Activity In Vitro

To infer the potential antioxidant activity (AC) of B, P, and PF juices, an adaptation of the 2,2-difenil-1-picrilhidrazil (DPPH) radical scavenging method [29,30] was followed. Fifty µL of each juice sample was filtered and diluted until absorbance values within the calibration curve were obtained. Juice samples, standards, and blank were mixed with 1.95 mL of methanolic DPPH 0.1 mM solution and kept at complete darkness for one hour at room temperature. Methanol instead of juice was used for the blank. The final mixture absorbance was measured using a spectrophotometer at 515 nm.

A Trolox five-point standard calibration curve (0.1–0.7 mM) was used for the quantification of antioxidant capacities, and the results were expressed as Trolox equivalent (TE) using units of µmols/L.

#### 2.2.5. Metabolomic Analyses

Metabolomic analyses of B, P, and PF juices were carried out to investigate the production of potential bioactive compounds by *L. plantarum* KABP051 during fermentation. Juice samples were centrifuged (14,000× *g*), filtered (0.2 µm), and stored at −20 °C until needed. Metabolomic analyses were performed by MS-Omics (Copenhagen, Denmark) as follows.

For the short chain fatty acids (SCFA) analyses, samples were acidified using hydrochloride acid, and deuterium labelled internal standards were added. Samples were analysed in a randomised order without extraction. Analysis was performed using a high polarity column (Zebron™ ZB-FFAP, GC Cap. Column 30 m × 0.25 mm × 0.25 μm) installed in a GC (7890B, Agilent, Madrid, Spain) coupled with a time-of-flight MS (Pegasus^®^ BT, LECO, Madrid, Spain). The system was controlled by ChromaTOF^®^ (LECO Instrumentos S.L., Madrid, Spain). Raw data were converted to netCDF format using Chemstation (Agilent), before the data were imported and processed in Matlab R2014b (Mathworks Inc., Natick, MA, USA) using the PARADISe software version 6 described by Johnsen et al. [31].

The semi-polar metabolite analysis was carried out using a Thermo Scientific Vanquish LC coupled to an Orbitrap Exploris 240 MS, Thermo Fisher Scientific (Madrid, Spain). Samples were extracted as follows: 75 μL samples were mixed with 25 μL aqueous solution of zink nitrate hexahydrate (5% *w*/*v*) and ammonium thiocyanate (20% *w*/*v*). After centrifugation at 15,000× *g* for 15 min at 4 °C, supernatant was filtered through a 0.22 μm filter. An electrospray ionisation interface was used as ionisation source. Analysis was performed in polarity switching ionisation mode.

The UPLC was performed using a slightly modified version of the protocol described by Doneanu et al. [32]. Peak areas were extracted using Compound Discoverer 3.2 (Thermo Scientific). The identification of compounds was performed at four levels; Level 1: identification by retention times (compared against in-house authentic standards), accurate mass (with an accepted deviation of 3 ppm), and MS/MS spectra; Level 2a: identification by retention times (compared against in-house authentic standards) and accurate mass (with an accepted deviation of 3 ppm); Level 2b: identification by accurate mass (with an accepted deviation of 3 ppm), and MS/MS spectra; and Level 3: identification by accurate mass alone (with an accepted deviation of 3 ppm).

Finally, a targeted metabolomic analysis following the same semi-polar metabolite method was conducted to verify the identity and the concentration of the following compounds: Nicotinic acid (CAS: 59-67-6), Phenyllactic acid (CAS: 828-01-3), Acetylcholine (CAS: 51-84-3) and N-Acetyl-L-glutamine (CAS: 2490-97-3).

#### 2.2.6. Consumer Acceptability

A sensory evaluation with 51 untrained panellists recruited amongst faculty staff and students (27 females and 24 males; aged 23–63 years old) was conducted to assess whether the average consumer could detect differences between the products. Prior to the tasting, the panellists were provided with detailed instructions regarding the product, the evaluation parameters, and the methodology to be followed during the assessment. Consumers were asked to evaluate the intensity and acceptability of different parameters as well as the purchase probability of fermented probiotic orange juice. Two different samples were tested: original orange juice without probiotic and orange juice with not less than 10^7^ probiotic CFU/mL fermented at 37 °C for 24 h.

The evaluation sessions took place 52 h after the inoculation and the juices were served at 5 ± 3 °C, poured in portions of 25 mL into white cups, identified with random three-digit codes and offered simultaneously to panellists. Bottled water was available to rinse the mouth between samples. A nine-point scale was used for the evaluation of acceptability and intensity of aroma, colour, bitterness, sweetness, acidity, and strange flavours, with 1 = Dislike extremely/Extremely low intensity and 9 = Like extremely/Extremely intense. The parameter “Purchase probability” was evaluated with a five-point scale, 1 = Definitely will not buy and 5 = Definitely will buy. The parameter “Overall preference” was evaluated by placing the three samples in order of preference: 1 = Worst; 2 = Intermediate; 3 = Best.

#### 2.2.7. Statistical Analysis

All experiments were carried out in triplicate, and values are reported as mean ± standard deviation (SD). Physicochemical, microbiological, and sensory evaluation tests were analysed using *t*-test. Metabolomic results were analysed using *t*-test, and the Benjamini–Hochberg correction was applied to control the false discovery rate (FDR = 0.1). The principal components analysis (PCA) models and their loading plots were calculated on the relative concentrations of the variables annotated on levels 1, and 2a.

Differences with a two-sided *p* value < 0.05 were considered statistically significant. Statistical analyses were performed with software PAST v4 (PCA and loading plots) and GraphPad PRISM v8 (all other analyses).

## 3. Results

### 3.1. Lactiplantibacillus plantarum KABP051 Stability in Supplemented and Fermented Orange, Apple and Peach Juices During 60 Days at 4 °C

**Figure 1:**
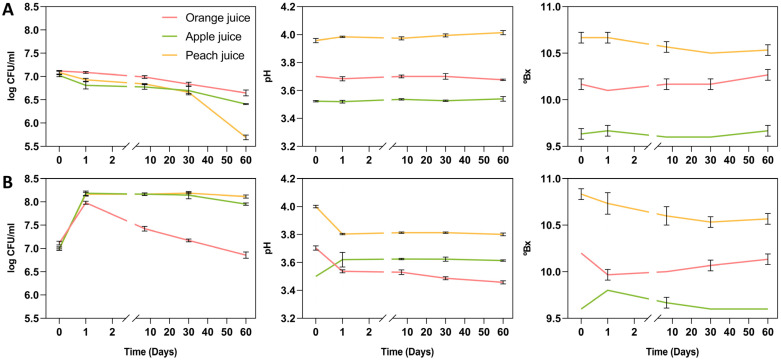
Results of stability analysis (log CFU/mL, pH and total soluble solids (°Bx)) in orange, apple, and peach juices inoculated with Lactiplantibacillus plantarum KABPTM 051 and stored 60 days at 4 °C without fermentation (**A**) and after a fermentation of 24 h at 37 *°C* (**B**).

The initial inoculum was calculated to reach the clinical dose (not less than 10^9^ CFU) per serving, defined as 240 mL/day of 100% fruit juices [33]. As shown in Figure 1A, CFU counts of orange and apple supplemented (non-fermented) juices maintained good stability throughout the study, while peach juice showed a remarkable reduction in bacterial viability after 4 weeks. Initial counts were 7.12 ± 0.00 log CFU/mL in orange juice, 7.02 ± 0.03 log CFU/mL in apple juice, and 7.08 ± 0.05 log CFU/mL in peach juice. After 60 days of cold storage, the bacterial load was 6.65 ± 0.06 log CFU/mL in orange juice, 6.41 ± 0.01 log CFU/mL in apple juice, and 5.69 ± 0.05 log CFU/mL in peach juice. No bacterial/fungal contamination was detected throughout the study. 

The initial pH values of orange, apple, and peach juices were 3.69 ± 0.01, 3.51 ± 0.01 and 3.95 ± 0.01, respectively. pH remained stable in all juices throughout the two months of storage (Figure 1A). The average of total soluble solids that initially measured 10.1 ± 0.1 °Bx in orange juice, 9.6 °Bx in apple and 10.7 ± 0.1 °Bx in peach juices showed a similar trend to pH, without significant changes throughout the study (Figure 1A).

In the 24 h-fermented samples (Figure 1B), the number of CFU/mL in all three juices was significantly higher than the initial inoculum. The initial average count was 7.11 ± 0.04 log CFU/mL in orange juice, 7.00 ± 0.04 log CFU/mL in apple juice, and 6.99 ± 0.02 log CFU/mL in peach juice. After fermentation, *L. plantarum* KABP051 concentration increased up to 7.98 ± 0.04 log CFU/mL in orange juice, 8.18 ± 0.05 log CFU/mL in apple juice, and 8.17 ± 0.04 log CFU/mL in peach juice. After 60 days of cold storage, CFU counts were 6.86 ± 0.07 log CFU/mL in orange juice, 7.95 ± 0.02 log CFU/mL in apple juice, and 8.11 ± 0.03 log CFU/mL in peach juice.

The pH of orange and peach juices significantly decreased after fermentation from 3.70 ± 0.02 to 3.54 ± 0.01 and from 4.00 ± 0.01 to 3.80 ± 0.01, respectively (both *p* ≤ 0.0001). In contrast, the pH of apple juice showed a slight but significant increase after fermentation from 3.50 to 3.62 ± 0.05 (*p* = 0.017). The pH of the three juices remained stable during the two months of cold storage. No major changes were noted for total soluble solids (TSS) in the three juices after fermentation, although a slight decrease was observed for orange and apple juices and a small increase was noted for the apple juice. These results indicate that *L. plantarum* KABP051 is stable and can ferment fruit juices, increasing CFU counts and modifying the pH of the matrix.

### 3.2. Total Phenolic Content and DPPH Radical Scavenging Activity

Table 1 shows the results of the total phenolic content (TPC) and DPPH radical scavenging activity of day 1 samples. Among blank samples, apple juice showed the lowest TPC, followed by peach juice and orange juice. After fermentation, only orange juice showed a TPC increment (from 709.9 ± 22.5 to 746.1 ± 27.6 mg GAE/L), although it did not reach statistical significance. The DPPH radical scavenging activity of blank juices correlated with their TPC, as the apple sample showed the lowest potential antioxidant capacity, followed by peach and orange juice. As for TPC results, no statistically significant differences in antioxidant potential were noted in fermented juices.

### 3.3. Metabolome Profiling of Fruit Juices

**Figure 2:**
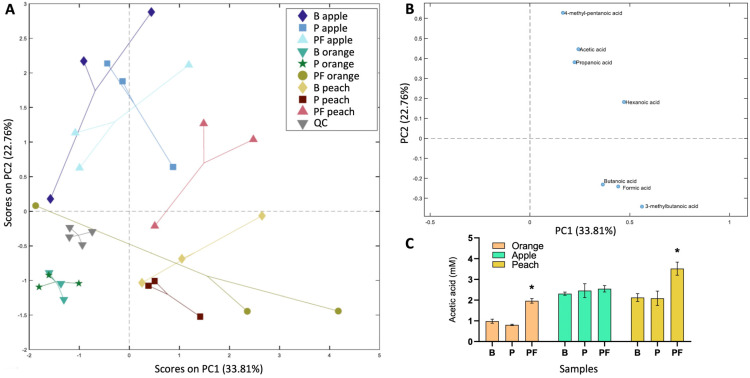
Metabolomic analysis of SCFAs in fruit juices. (**A**) Score plot of PC2 over PC1 in the model calculated on the relative concentrations of SCFAs in the reduced dataset. Data have been autoscaled. (**B**) Loading plot from PCA model calculated on the relative concentrations of SCFAs in the reduced dataset. Data have been autoscaled, and overlapping labels were removed to improve readability. (**C**) Acetic acid concentration (mM) in blank (B), probiotic-supplemented (P), and probiotic-fermented (PF) fruit juices. * *p* < 0.05.

To further characterise the functionality of fruit juices, metabolomic analyses were conducted in day 1 samples. First, the presence of short-chain fatty acids (SCFA) was investigated by targeted approaches (Figure 2). Formic acid was the most abundant SCFA in blank juices at around 10, 7, and 12 mM concentration in orange, apple, and peach juices, respectively (Table S1). Acetic acid was also detected in the three juices at lower concentration: around 1, 2, and 2 mM in orange, apple, and peach juices, respectively. The other SCFAs analysed were found at very low concentrations. The presence of probiotic did not modify the concentration of any of the SCFAs, but probiotic-assisted fermentation significantly increased levels of acetic acid in orange (from 0.98 ± 0.1 mM to 1.96 ± 0.11 mM) and peach (from 2.12 ± 0.19 mM to 3.51 ± 0.32 mM) juices (Figure 2C).

**Figure 3:**
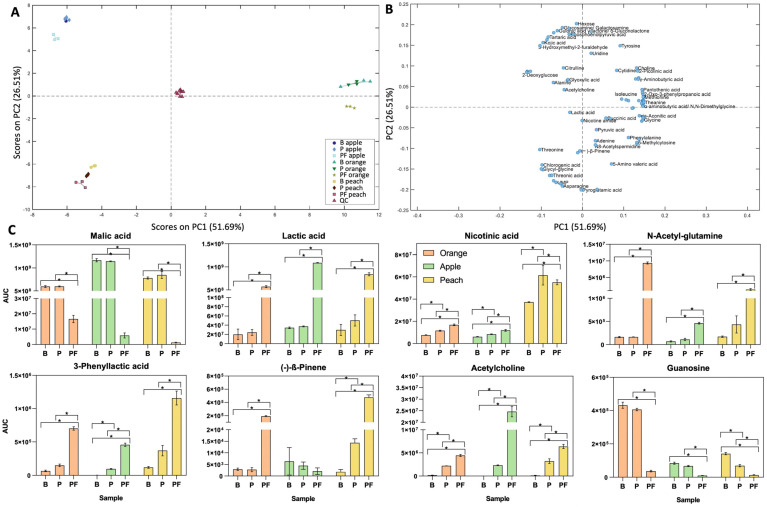
Semi-polar metabolite profile. (**A**) Score plot of PC2 over PC1 in the model calculated on the relative concentrations of the variables annotated on levels 1 and 2a in the reduced dataset. (**B**) Loading plot from PCA model calculated on the relative concentrations of the variables annotated on levels 1 and 2a in the reduced dataset. Data have been autoscaled, and overlapping labels were removed to improve readability. (**C**) Relative abundances (Area Under the Curve) of compounds of interest detected in level 1 and 2a in blank (B), probiotic-supplemented (P), and probiotic-fermented (PF) fruit juices. * *p* < 0.05.

An untargeted semi-polar metabolite analysis was also conducted to identify additional beneficial compounds produced by *L. plantarum* KABP051 during the fermentation of fruit juices. A total of 672 compounds were detected, of which 32 metabolites could be identified as level 1; 56 compounds were annotated as level 2a; 21 were annotated as level 2b, and, with less certainty, 128 compounds were detected in level 3. Finally, 439 compounds were distinguished from the background but remained unknown. The first two principal components explained 51.69% (PC1) and 26.51% (PC2) of the total variation (Figure 3A,B).

The presence of *L. plantarum* KABP051 in fruit juices, with or without fermentation, modified the concentration of 22 compounds (Table S2). Eleven compounds significantly increased in some of the P or PF samples, while the concentration of the rest of the compounds decreased, evidencing some metabolic pathways. For instance, in PF samples, the concentration of malic acid drastically decreased in favour of lactic acid (Figure 3C), confirming that malic acid is a major carbon source for *L. plantarum* KABP051 growth. Likewise, nicotinamide decreased while nicotinic acid increased in PF samples, indicating that *L. plantarum* KABP051can modify vitamin B forms, changing the vitamin profile of the juices. Moreover, probiotic fermentation raised the concentration of additional bioactive compounds including n-acetyl glutamine (NAG), phenyllactic acid (PLA), (−)-β-pinene, and acetylcholine (ACh) (Figure 3C), among others (Table S2). In addition to malic acid, the nucleosides adenosine, guanosine, and cytidine were amongst the top reduced compounds in juices supplemented with *L. plantarum* KABP051 (Table S2).

The precise concentration of nicotinic acid, ACh, PLA, and NAG was determined by targeted metabolomic analysis (Table 2). These results validated those observed for the untargeted analyses (Figure 3C) and confirmed that *L. plantarum* KABP051 can significantly synthesise these biomolecules at µM range. While nicotinic acid, ACh, and PLA were already present at detectable concentrations in the blank juices, the amino acid NAG was not detected in any blank sample but was produced during fermentation in the three juices, up to a maximum of 11.26 ± 0.39 µM in orange juice.

### 3.4. Sensory Evaluation

A total of 51 independent panellists were enrolled to evaluate the organoleptic properties (intensity and acceptability) of the original orange juice (Blank; B) and the fermented version (PF).

**Figure 4:**
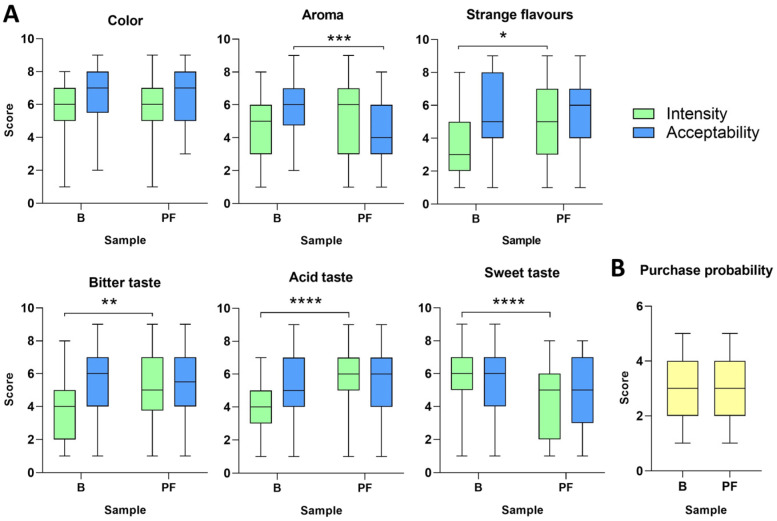
Results of the sensory evaluation involving 51 untrained panellists. (**A**) Results of organoleptic parameters and (**B**) score of the purchase probability of blank juices (**B**) and probiotic-fermented (PF) orange juices. A nine-point scale was used for the evaluation of acceptability and intensity of aroma, colour, bitterness, sweetness, acidity, and strange flavours, with 1 = Dislike extremely/Extremely low intensity and 9 = Like extremely/Extremely intense. The parameter “Purchase probability” was evaluated with a five-point scale, with 1 = Definitely will not buy and 5 = Definitely will buy. * *p* < 0.05, ** *p* < 0.01, *** *p* < 0.001, **** *p* < 0.0001.

As shown in Figure 4, probiotic fermentation did not alter colour perception by panellists. The intensity of the aroma was not significantly altered in probiotic-fermented juice, but its acceptability was significantly reduced. Strange flavour perception significantly increased in fermented samples without affecting its acceptability. Although a significant increase in bitter and acid intensity was noted for the fermented version, it did not translate into a reduced acceptability. The intensity of sweet taste significantly decreased in fermented juices, but it did not affect acceptance scores. Finally, and in line with sensory results, similar purchase probability was declared by participants between original and 24 h-fermented orange juices (Figure 4B). These results indicate that, although fermentation for 24 h affected different organoleptic parameters, overall acceptance is comparable to the original orange juice.

## 4. Discussion

The fermentation of fruit and vegetable juices by well-documented probiotics represents a promising nutritional approach for the development of novel non-dairy functional beverages. It is paramount to select the best probiotic strains that combine excellent in vitro, in vivo, and clinical evidence with an adequate stability. *Lactiplantibacillus plantarum* is one of the most used species for vegetable fermentation [34], likely because of its metabolic versatility and environmental resilience. Our results demonstrate that probiotic strain *L. plantarum* KABP051 displays an excellent viability in orange, apple, and peach juices, both in probiotic-fermented and non-fermented forms, throughout 60 days of cold storage. Only the non-fermented peach sample displayed probiotic CFU counts below the recommended 10^6^–10^7^ CFU/mL dose [19,35] at day 60, indicating this is the most unfavourable condition for probiotic stability tested in our study. Nonetheless, this problem could be solved by increasing the initial inoculum. Interestingly, probiotic fermentation increased CFU counts of *L. plantarum* KABP051 in all juices, reaching CFUs per serving of 240 mL [33] higher than the therapeutic dose (>10^9^ CFUs) [23,24] in the three fermented juices for up to 60 days of cold storage (Figure 1B), thus qualifying as “probiotic fermented foods” [12]. These results suggest that the *L. plantarum* KABP051 fermentation of fruit juices might reduce probiotic production costs while maximising its viability and health benefits.

Different studies have investigated the stability of some probiotic strains in non-fermented fruit juices. For instance, the popular probiotics *L. rhamnosus* GG (ATCC53103), *L. casei* DN-114 001, and *B. lactis* Bb-12 as well as *L. paracasei* NFBC43338 showed good survival in orange and pineapple juices, with CFUs above the recommended dose for up to 12 weeks under refrigeration [36]. On the contrary, *L. salivarius* strains UCC118 and UCC500 showed very poor viability (<10^6^ CFU/mL) in orange and pineapple juices after only two weeks [36].

Regarding probiotic viability in fermented fruit juices, it was found that *L. casei* NRRL B442 was able to survive (>10^6^ CFU/mL) in fermented pineapple juice for 42 days in refrigerated conditions, while the addition of sucrose reduced *L. casei* NRRL B442 viability to 28 days [37]. Interestingly, when *L. casei* NRRL B442 was used to ferment cashew apple juice, high CFU counts (>10^8^ CFU/mL) were recorded throughout the storage period of 42 days at 4 °C [38]. Likewise, Dimitrovski et al. [39] also found apple juice to be a suitable matrix for probiotic fermentation and viability using *L. plantarum* strain PCS 26. Our results are in accordance with previous studies, confirming that apple juice is an excellent medium for the growth and stability of *L. plantarum* KABP051, as fermented apple samples maintained ~10^8^ CFU/mL after 60 days of refrigerated storage. While similar counts were noted in the fermented peach sample, a more marked CFU reduction was observed in the fermented orange samples at the end of the study. Taken together, probiotic viability in fermented juices seems to be significantly influenced by many aspects including the composition of the matrix and the strain specificity. Therefore, probiotic stability in fruit juices cannot be taken for granted and should be determined on a case-by-case basis.

While the pH of fermented orange and peach juices decreased compared to unfermented and control juices, the pH of fermented apple juice, where malic acid is the most abundant organic acid [40], showed a slight increment. This can be explained by the malolactic fermentation (transformation of malic acid into lactic acid), as lactic acid (pKa = 3.86) is a weaker acid than malic acid (pKa1 = 3.40) and therefore, reduction of malic acid in favor of lactic acid translates into a slight increase of apple juice pH [41].

In a previous study, Yuasa et al. [42] reported that *L. plantarum* SI-1 and *L. pentosus* MU-1 exclusively utilise L-malic acid to produce lactic acid, without affecting total sugar concentration of citric juices. Our results confirm this notion and show that malolactic fermentation is the main energy metabolic pathway of strain KABP051 not only in orange and apple but also in peach juices.

TPC and antioxidant (DPPH-RSA) capacity remained unaltered in all fermented and unfermented juices (Table 1). Although an increase in TPC and DPPH-RSA was noted for the probiotic-fermented orange juice, it did not reach statistical significance. In this line, only the flavanones naringin and hesperidin, but not their aglycone forms, were detected in orange juice. Probiotic introduction and fermentation did not modify flavanone’s profile. In a previous work, de la Fuente et al. [43] showed that *L. brevis* strain POM and *L. plantarum* strains TR-71 and TR-14 were able to ferment orange-juice milk beverages resulting in increased antioxidant capacity, which was in part attributed to the production of phenyllactic acid (PLA) by lactobacilli. Although we also detected a great increase in the phenolic compound PLA after probiotic fermentation, it did not translate into a significant increase in TPC and antioxidant capacity. Of note, de la Fuente et al. [43] conducted a 72 h fermentation in milk-based beverages with different conditions from those assessed in our work, which may explain discrepancies in antioxidant and TPC results.

In order to identify metabolic products of interest, different metabolomic approaches were performed. The results showed that the fermentation not only increased the probiotic biomass but also allowed for the production of molecules of medical, nutraceutical, and biotechnological interest. The short-chain fatty acid (SCFA) profile was enhanced with a significant increment in the acetic acid content in all fermented beverages. SCFAs play an important role in human health; therefore, introducing a fortified food, rich in these compounds, could contribute to maintaining a correct endogenous SCFA balance [44]. Acetic acid has antioxidant, antibacterial, anti-diabetic, and anti-tumour properties and prevents cardiovascular disorders [45,46]. Moreover, acetic acid in the gut is the preferred substrate for the butyrate-producing bacteria *Roseburia* spp. and *Faecalibacterium prausnitzii* to produce butyric acid, the major carbon source of enterocytes [47].

Semi-polar metabolite profiling confirmed malolactic fermentation, typically used in wine production [48], and revealed the production of several additional bioactive compounds. Among the common compounds produced by the probiotic fermentation of all juices, nicotinic acid, acetylcholine (ACh), phenyllactic acid (PLA), and n-acetyl glutamine (NAG) were verified and quantified by targeted approaches (Table 2).

Nicotinic acid is a form of vitamin B3 and is an essential human nutrient. According to EFSA [49], dietary reference values for niacin range between 13 to 19 mg/day for adults. Among the juices investigated, nicotinic acid was particularly high in peach (1.7 mg/L), and the presence of the probiotic significantly increased its concentration up to ~2.4 mg/L. Some probiotic lactobacilli have been reported to produce a number of vitamins belonging to the B-complex, such as folate, vitamin B12, and thiamine [50,51]. However, to our knowledge, this is the first time that the synthesis of niacin during fruit juice fermentation by *L. plantarum* has been described.

Acetylcholine is a neurotransmitter that has a wide variety of functions in the brain and other organs. ACh production has been described in many gram-negative and gram-positive bacteria including lactobacilli [52]. It is well known that some lactobacilli can produce ACh [53], but to our knowledge this is the first time that a *L. plantarum* strain has shown the ability to synthesise this neurotransmitter in orange, peach, and apple juices. Intestinal ACh exerts a localised immunomodulatory effect [54] and can improve intestinal barrier function [53,55]. The highest levels of ACh were found in the probiotic-fermented apple juice.

PLA is typically produced during fermentation of dairy products [56] but has been also detected in supernatants of different *Lactobacillus* spp. strains grown in the presence of pomegranate extract [57] and in fermented orange juices containing milk [43]. It has demonstrated broad spectrum antimicrobial activity against spoilage organisms and pathogens, both bacteria and fungi, which prolongs fermented food shelf life [56,58]. PLA content drastically increased in all probiotic-fermented juices, peach juice being the most concentrated sample.

Glutamine is very abundant in the human body, but it is unstable in liquid media, and N-acetyl-l-glutamine (NAG) has been used as supplement to avoid instability and intolerance issues [59]. Different clinical studies have reported that enteral or parenteral nutrition formulas containing this amino acid help maintain the structural and functional integrity of the gut [60,61]. Supplements containing glutamine forms are widely used in sports nutrition, especially because of its immunomodulatory role, but recent studies have also evidenced some improvement in fatigue markers including increased glycogen synthesis and reduced ammonia accumulation [62]. Interestingly, NAG was only detected in probiotic-fermented juices, while its concentration in probiotic-supplemented and blank samples was below the limit of detection. These results suggest that fermentation assisted by *L. plantarum* KABP051 de novo produces this interesting amino acid, which was absent from original juices.

Besides the production of interesting bioactive compounds, the fermentation of fruit juices also evidenced the ability of *L. plantarum* KABP051 to consume some nutrients (Figure 3 and Table S2). Of note, the probiotic consumed the purines adenosine, guanosine, and cytidine in all fermented juices. Purines are the major substrate for uric acid formation and crystallisation, which could lead to gout [63]. Therefore, the consumption of a probiotic that has the ability to reduce dietary purines in the gut could be of interest for patients prone to hyperuricemia [64], as reported for *Lactobacillus gasseri* strain PA-3 [65].

For the sensory evaluation, orange juice was selected because it is the most popular and consumed juice worldwide [66] and Spain is the leading orange producer in the EU, according to USDA (2020). A sensory evaluation study was conducted to gain insights into consumers’ perception and acceptability. The fermentation of orange juice by the probiotic KABP051 only reduced aroma acceptability and increased the intensity of strange flavours and bitter and acid taste, whereas that of sweet taste was reduced. Similarly, Yuasa et al. [42] reported that the fermentation of mandarin and orange juices by *L. plantarum* SI-1 increased sourness and acidic bitterness due to an increase in lactic acid and total organic acids, which is also observed in our analyses.

The present study has certain limitations. First, as an attempt to evaluate the functionality of probiotic juices, DPPH-RSA capacity and TPC experiments were performed, and results showed no differences compared to commercial beverages. Therefore, although *L. plantarum* KABP051 has shown probiotic effects in several trials [23,24,25,26], future in vitro, in vivo, or clinical studies are required to confirm that fermented fruit juices retain and improve probiotic properties. Second, as proof of concept, only probiotic fermented orange juices were assessed in a sensory evaluation study. Thus, additional sensory experiments are required for peach and apple fermented beverages to confirm that their organoleptic parameters and overall acceptance fit consumer tastes. Finally, the metabolomic analyses performed in our study were not suitable for the detection of medium- and long-chain fatty acids (LCFA), and therefore it is unknown whether *L. plantarum* KABP051 metabolism could influence the composition of these relevant compounds. Future targeted approaches should be conducted to elucidate if probiotic fermentation may improve the profile of LCFA, such as conjugated linolenic acid, which displays remarkable beneficial effects [67].

In conclusion, we here show that fermentation of fruit juices by *L. plantarum* KABP051 may result in tasty functional probiotic juices that are enriched with a number of health-promoting compounds. Thus, the development of these functional juices may have a dual function to the host: the delivery of a well-characterised probiotic combined with fruit juices with increased nutritional value.

## Figures and Tables

**Table 1:** Total phenolic compounds (TPC) and DPPH radical scavenging activity (DPPH-RSA) of orange, apple, and peach juices.

	Orange Juice		Apple Juice		Peach Juice	
	TPC	DPPH-RSA	TPC	DPPH-RSA	TPC	DPPH-RSA
	(Gallic Acid mg/L)	(Trolox µmols/L)	(Gallic Acid mg/L)	(Trolox µmols/L)	(Gallic Acid mg/L)	(Trolox µmols/L)
**B**	709.9 ± 22.5	2309 ± 127.5	262.8 ± 21.3	137.7 ± 16.1	314.4 ± 2.3	783.1 ± 4.9
**P**	682.3 ± 2.9	2722.9 ± 131.4	264.8 ± 1.1	136.4 ± 31.5	320.5 ± 23.6	782.2 ± 18.5
**PF**	746.1 ± 27.6	2731.3 ± 190.3	269.7 ± 14.9	139.5 ± 59.5	312.4 ± 8.6	790.8 ± 23.6

DPPH: 2,2-difenil-1-picrilhidrazil. B: Blank; P: probiotic (non-fermented); PF: probiotic fermented.

**Table 2:** Concentration (average ± SD) in µM of Nicotinic acid, Acetylcholine, Phenyllactic acid, and N-Acetyl-L-Glutamine detected in fruit samples by targeted metabolomics.

	Apple			Orange			Peach		
	B	P	PF	B	P	PF	B	P	PF
Nicotinic acid	1.56 ± 0.10	2.36 ± 0.03 *	3.42 ± 0.09 *	2.42 ± 0.06	3.9 ± 0.22 *	5.85 ± 0.09 *	13.82 ± 0.17	20.21 ± 0.37 *	19.24 ± 0.51 *
Acetylcholine	0.09 ± 0.00	0.29 ± 0.00 *	2.41 ± 0.11 *	0.09 ± 0.00	0.29 ± 0.01 *	0.51 ± 0.01 *	0.09 ± 0.00	0.32 ± 0.00 *	0.64 ± 0.02 *
Phenyllactic acid	0.23 ± 0.04	1.32 ± 0.01 *	5.16 ± 0.15 *	0.5 ± 0.05	1.34 ± 0.05 *	7.57 ± 0.29 *	1.56 ± 0.07	3.68 ± 0.24 *	12.04 ± 0.40 *
N-Acetyl-L-Glutamine	<LOD	<LOD	0.3 ± 0.01 *	<LOD	<LOD	11.26 ± 0.39 *	<LOD	0.16 ± 0.02	1.28 ± 0.19 *

B: Blank; P: probiotic (non-fermented); PF: probiotic fermented. LOD: Limit Of Detection. * *p* < 0.05; *t*-test PF vs. B.

## Data Availability

The original contributions presented in the study are included in the article/supplementary material, further inquiries can be directed to the corresponding author.

## Supplementary Materials

The following supporting information can be downloaded at: https://www.mdpi.com/article/10.3390/foods13233851/s1, Figure S1: Schematic representation of the experimental design; Table S1: Concentration of SCFA (mM) in fruit juices; Table S2: Additional compounds detected, consumed or produced by *L. plantarum* KABP051 in fruit juices.

## Abbreviations

The following abbreviations are used in this manuscript:

CFU	colony forming units
P	probiotic
PF	probiotic-fermented
B	blank
ISAPP	International Scientific Association for Probiotics and Prebiotics
MRS	de Man, Rogosa, and Sharpe
TSS	total soluble solids
TPC	total phenolic content
GAE	gallic acid equivalent
TE	trolox equivalent
AC	antioxidant activity
DPPH	2,2-difenil-1-picrilhidrazil
NAG	n-acetyl glutamine
PLA	phenyllactic acid
ACh	acetylcholine
FDR	false discovery rate
PCA	principal components analysis
SCFA	short-chain fatty acids

## References

[1] Rajilic-Stojanovic M., Figueiredo C., Smet A., Hansen R., Kupcinskas J., Rokkas T., Andersen L., Machado J.C., Ianiro G., Gasbarrini A. (2020). Systematic review: Gastric microbiota in health and disease. Aliment. Pharmacol. Ther..

[2] Hou K., Wu Z.X., Chen X.Y., Wang J.Q., Zhang D., Xiao C., Zhu D., Koya J.B., Wei L., Li J. (2022). Microbiota in health and diseases. Signal Transduct. Target. Ther..

[3] Bauer K.C., Littlejohn P.T., Ayala V., Creus-Cuadros A., Finlay B.B. (2022). Nonalcoholic Fatty Liver Disease and the Gut-Liver Axis: Exploring an Undernutrition Perspective. Gastroenterology.

[4] Dumas A., Bernard L., Poquet Y., Lugo-Villarino G., Neyrolles O. (2018). The role of the lung microbiota and the gut–lung axis in respiratory infectious diseases. Cell. Microbiol..

[5] Järbrink-Sehgal E., Andreasson A. (2020). The gut microbiota and mental health in adults. Curr. Opin. Neurobiol..

[6] Mancin L., Wu G.D., Paoli A. (2023). Gut microbiota–bile acid–skeletal muscle axis. Trends Microbiol..

[7] Morais L.H., Schreiber H.L., Mazmanian S.K. (2020). The gut microbiota–brain axis in behaviour and brain disorders. Nat. Rev. Microbiol..

[8] Szántó M., Dózsa A., Antal D., Szabó K., Kemény L., Bai P. (2019). Targeting the gut-skin axis—Probiotics as new tools for skin disorder management?. Exp. Dermatol..

[9] Ahlawat S., Asha, Sharma K.K. (2021). Gut–organ axis: A microbial outreach and networking. Lett. Appl. Microbiol..

[10] Shimizu M. (2014). History and Current Status of Functional Food Regulations in Japan. Nutraceutical and Functional Food Regulations in the United States and Around the World.

[11] Hill C., Guarner F., Reid G., Gibson G.R., Merenstein D.J., Pot B., Morelli L., Canani R.B., Flint H.J., Salminen S. (2014). Expert consensus document. The International Scientific Association for Probiotics and Prebiotics consensus statement on the scope and appropriate use of the term probiotic. Nat. Rev. Gastroenterol. Hepatol..

[12] Marco M.L., Sanders M.E., Gänzle M., Arrieta M.C., Cotter P.D., De Vuyst L., Hill C., Holzapfel W., Lebeer S., Merenstein D. (2021). The International Scientific Association for Probiotics and Prebiotics (ISAPP) consensus statement on fermented foods. Nat. Rev. Gastroenterol. Hepatol..

[13] Binda S., Hill C., Johansen E., Obis D., Pot B., Sanders M.E., Tremblay A., Ouwehand A.C. (2020). Criteria to Qualify Microorganisms as “Probiotic” in Foods and Dietary Supplements. Front. Microbiol..

[14] Boethius A. (2016). Something rotten in Scandinavia: The world’s earliest evidence of fermentation. J. Archaeol. Sci..

[15] Jeong C., Wilkin S., Amgalantugs T., Bouwman A.S., Taylor W.T.T., Hagan R.W., Bromage S., Tsolmon S., Trachsel C., Grossmann J. (2018). Bronze Age population dynamics and the rise of dairy pastoralism on the eastern Eurasian steppe. Proc. Natl. Acad. Sci..

[16] Perruchini E., Glatz C., Hald M.M., Casana J., Toney J.L. (2018). Revealing invisible brews: A new approach to the chemical identification of ancient beer. J. Archaeol. Sci..

[17] Leeuwendaal N.K., Stanton C., O’toole P.W., Beresford T.P. (2022). Fermented Foods, Health and the Gut Microbiome. Nutrients.

[18] Şanlier N., Gökcen B.B., Sezgin A.C. (2019). Health benefits of fermented foods. Crit. Rev. Food Sci. Nutr..

[19] Mojikon F.D., Kasimin M.E., Molujin A.M., Gansau J.A., Jawan R. (2022). Probiotication of Nutritious Fruit and Vegetable Juices: An Alternative to Dairy-Based Probiotic Functional Products. Nutrients.

[20] Maia M.S., Domingos M.M., de São José J.F.B. (2023). Viability of Probiotic Microorganisms and the Effect of Their Addition to Fruit and Vegetable Juices. Microorganisms.

[21] Mayo B., Fiórez A.B. (2022). Lactic Acid Bacteria: Lactobacillus plantarum. Encyclopedia of Dairy Sciences.

[22] Martino M.E., Bayjanov J.R., Caffrey B.E., Wels M., Joncour P., Hughes S., Gillet B., Kleerebezem M., van Hijum S.A.F.T., Leulier F. (2016). Nomadic lifestyle of *Lactobacillus plantarum* revealed by comparative genomics of 54 strains isolated from different habitats. Environ. Microbiol..

[23] Chong H.X., Yusoff N.A.A., Hor Y.Y., Lew L.C., Jaafar M.H., Choi S.B., Yusoff M.S., Wahid N., Abdullah M.F.I., Zakaria N. (2019). *Lactobacillus plantarum* DR7 improved upper respiratory tract infections via enhancing immune and inflammatory parameters: A randomized, double-blind, placebo-controlled study. J. Dairy Sci..

[24] Chong H.X., Yusoff N.A.A., Hor Y.Y., Lew L.C., Jaafar M.H., Choi S.B., Yusoff M.S.B., Wahid N., Bin Abdullah M.F.I.L., Zakaria N. (2019). *Lactobacillus plantarum* DR7 alleviates stress and anxiety in adults: A randomised, double-blind, placebo-controlled study. Benef. Microbes..

[25] Rizzi F., Altadill T., Asto E., Perez M., Espadaler-Mazo J., Huedo P. (2024). Abstract 1339 Genetic and Phenotypic Equivalence Between Two Strains of *L. plantarum* Isolated in Separate Parts of the Planet Supports the Nomadism of Some Lactic Acid Bacteria. J. Biol. Chem..

[26] Nart J., Jiménez-Garrido S., Ramírez-Sebastià A., Astó E., Buj D., Huedo P., Espadaler J. (2021). Oral colonization *by Levilactobacillus brevis* KABP TM-052 and *Lactiplantibacillus plantarum* KABP TM-051: A Randomized, Double-Blinded, Placebo-Controlled Trial (Pilot Study). J. Clin. Exp. Dent..

[27] Magwaza L.S., Opara U.L. (2015). Analytical methods for determination of sugars and sweetness of horticultural products-A review. Sci. Hortic..

[28] Singleton V.L., Orthofer R., Lamuela-Raventós R.M. (1999). Analysis of total phenols and other oxidation substrates and antioxidants by means of folin-ciocalteu reagent. Methods Enzymol..

[29] Suárez-Jacobo Á., Rüfer C.E., Gervilla R., Guamis B., Roig-Sagués A.X., Saldo J. (2011). Influence of ultra-high pressure homogenisation on antioxidant capacity, polyphenol and vitamin content of clear apple juice. Food Chem..

[30] Sun J., Zhao C., Pu X., Li T., Shi X., Wang B., Cheng W. (2022). Flavor and Functional Analysis of *Lactobacillus plantarum* Fermented Apricot Juice. Fermentation.

[31] Johnsen L.G., Skou P.B., Khakimov B., Bro R. (2017). Gas chromatography—Mass spectrometry data processing made easy. J. Chromatogr. A.

[32] Doneanu C.E., Chen W., Mazzeo J.R. (2019). UPLC/MS Monitoring of Water-Soluble Vitamin Bs in Cell Culture Media in Minutes.

[33] Auerbach B.J., Dibey S., Vallila-Buchman P., Kratz M., Krieger J. (2018). Review of 100% Fruit Juice and Chronic Health Conditions: Implications for Sugar-Sweetened Beverage Policy. Adv. Nutr..

[34] Ayed L., M’Hir S., Hamdi M. (2020). Microbiological, Biochemical, and Functional Aspects of Fermented Vegetable and Fruit Beverages. J. Chem..

[35] Manoj P.M., Mohan J.R., Khasherao B.Y., Shams R., Dash K.K. (2023). Fruit based probiotic functional beverages: A review. J. Agric. Food Res..

[36] Sheehan V.M., Ross P., Fitzgerald G.F. (2007). Assessing the acid tolerance and the technological robustness of probiotic cultures for fortification in fruit juices. Innov. Food Sci. Emerg. Technol..

[37] Costa M.G.M., Fonteles T.V., De Jesus A.L.T., Rodrigues S. (2013). Sonicated pineapple juice as substrate for *L. casei* cultivation for probiotic beverage development: Process optimisation and product stability. Food Chem..

[38] Pereira A.L.F., Maciel T.C., Rodrigues S. (2011). Probiotic beverage from cashew apple juice fermented with *Lactobacillus casei*. Food Res. Int..

[39] Dimitrovski D., Velickova E., Langerholc T., Winkelhausen E. (2015). Apple juice as a medium for fermentation by the probiotic *Lactobacillus plantarum* PCS 26 strain. Ann. Microbiol..

[40] Blanco D., Quintanilla M.E., Mangas J.J., Gutierrez M.D. (2006). Determination of Organic Acids in Apple Juice by Capillary Liquid Chromatography. J. Liq. Chromatogr. Relat. Technol..

[41] Liu S.Q. (2002). Malolactic fermentation in wine—Beyond deacidification. J. Appl. Microbiol..

[42] Yuasa M., Shimada A., Matsuzaki A., Eguchi A., Tominaga M. (2021). Chemical composition and sensory properties of fermented citrus juice using probiotic lactic acid bacteria. Food Biosci..

[43] de la Fuente B., Luz C., Puchol C., Meca G., Barba F.J. (2021). Evaluation of fermentation assisted by *Lactobacillus brevis* POM, and *Lactobacillus plantarum* (TR-7, TR-71, TR-14) on antioxidant compounds and organic acids of an orange juice-milk based beverage. Food Chem..

[44] Xu Y., Zhu Y., Li X., Sun B. (2020). Dynamic balancing of intestinal short-chain fatty acids: The crucial role of bacterial metabolism. Trends Food Sci. Technol..

[45] Budak N.H., Aykin E., Seydim A.C., Greene A.K., Guzel-Seydim Z.B. (2014). Functional Properties of Vinegar. J. Food Sci..

[46] Chen G.L., Zheng F.J., Lin B., Yang Y.X., Fang X.C., Verma K.K., Yang L.F. (2023). Vinegar: A potential source of healthy and functional food with special reference to sugarcane vinegar. Front. Nutr..

[47] Duncan S.H., Holtrop G., Lobley G.E., Calder A.G., Stewart C.S., Flint H.J. (2004). Contribution of acetate to butyrate formation by human faecal bacteria. Br. J. Nutr..

[48] Fu J., Wang L., Sun J., Ju N., Jin G. (2022). Malolactic Fermentation: New Approaches to Old Problems. Microorganisms.

[49] Agostoni C., Berni Canani R., Fairweather-Tait S., Heinonen M., Korhonen H., La Vieille S., Marchelli R., Martin A., Naska A., Neuhäuser-Berthold M. (2014). Scientific Opinion on Dietary Reference Values for niacin. EFSA J..

[50] Chugh B., Kamal-Eldin A. (2020). Bioactive compounds produced by probiotics in food products. Curr. Opin. Food Sci..

[51] Masuda M., Ide M., Utsumi H., Niiro T., Shimamura Y., Murata M. (2012). Production Potency of Folate, Vitamin B12, and Thiamine by Lactic Acid Bacteria Isolated from Japanese Pickles. Biosci. Biotechnol. Biochem..

[52] Chen Y., Xu J., Chen Y. (2021). Regulation of Neurotransmitters by the Gut Microbiota and Effects on Cognition in Neurological Disorders. Nutrition.

[53] Perez M., Astó E., Huedo P., Alcántara C., Buj D., Espadaler J. (2020). Derived Postbiotics of a Multi-strain Probiotic Formula Clinically Validated for the Treatment of Irritable Bowel Syndrome. FASEB J..

[54] Wang H., Yu M., Ochani M., Amelia C.A., Tanovic M., Susarla S., Li J.H., Wang H., Yang H., Ulloa L. (2002). Nicotinic acetylcholine receptor α7 subunit is an essential regulator of inflammation. Nature.

[55] Yong S.J., Tong T., Chew J., Lim W.L. (2020). Antidepressive Mechanisms of Probiotics and Their Therapeutic Potential. Front. Neurosci..

[56] Valerio F., Lavermicocca P., Pascale M., Visconti A. (2004). Production of phenyllactic acid by lactic acid bacteria: An approach to the selection of strains contributing to food quality and preservation. FEMS Microbiol. Lett..

[57] Chamberlain M.C., O’Flaherty S., Cobián N., Barrangou R. (2022). Metabolomic Analysis of *Lactobacillus acidophilus*, *L. gasseri, L. crispatus*, and *Lacticaseibacillus rhamnosus* Strains in the Presence of Pomegranate Extract. Front. Microbiol..

[58] Lavermicocca P., Valerio F., Evidente A., Lazzaroni S., Corsetti A., Gobbetti M. (2000). Purification and characterization of novel antifungal compounds from the sourdough *Lactobacillus plantarum* strain 21B. Appl. Env. Microbiol..

[59] Arnaud A., Ramírez M., Baxter J.H., Angulo A.J. (2004). Absorption of enterally administered N-acetyl-l-glutamine versus glutamine in pigs. Clin. Nutr..

[60] Houdijk A.P.J., Rijnsburger E.R., Jansen J., Wesdorp R., Kweiss J., Mc Camish M.A., Teerlink T., Meuwissen S.G., Haarman H.J., Thijs L.G. (1998). Randomised trial of glutamine-enriched enterai nutrition on infectious morbidity in patients with multiple trauma. Lancet.

[61] Jones C., Allan Palmer T.E., Griffiths R.D. (1999). Randomized clinical outcome study of critically ill patients given glutamine-supplemented enteral nutrition. Nutrition.

[62] Coqueiro A.Y., Rogero M.M., Tirapegui J. (2019). Glutamine as an Anti-Fatigue Amino Acid in Sports Nutrition. Nutrients.

[63] Aihemaitijiang S., Zhang Y., Zhang L., Yang J., Ye C., Halimulati M., Zhang W., Zhang Z. (2020). The Association between Purine-Rich Food Intake and Hyperuricemia: A Cross-Sectional Study in Chinese Adult Residents. Nutrients.

[64] Wang Z., Li Y., Liao W., Huang J., Liu Y., Li Z., Tang J. (2022). Gut microbiota remodeling: A promising therapeutic strategy to confront hyperuricemia and gout. Front. Cell Infect. Microbiol..

[65] Yamada N., Iwamoto C., Kano H., Yamaoka N., Fukuuchi T., Kaneko K., Asami Y. (2016). Evaluation of purine utilization by *Lactobacillus gasseri* strains with potential to decrease the absorption of food-derived purines in the human intestine. Nucleosides Nucleotides Nucleic Acids.

[66] Pan X., Bi S., Lao F., Wu J. (2023). Factors affecting aroma compounds in orange juice and their sensory perception: A review. Food Res. Int..

[67] Li S., Xu L., Qing J., Wu X., Li H., Chen H., Liu X. (2023). Multiple biological activities and biosynthesis mechanisms of specific conjugated linoleic acid isomers and analytical methods for prospective application. Food Chem..

