# Supplementary material for: Lactiplantibacillus plantarum KABP051: Stability in Fruit Juices and Production of Bioactive Compounds During Their Fermentation
Source: Foods. 2024 Nov 28;13(23):3851. doi: 10.3390/foods13233851 (PMC11640170; doi:10.3390/foods13233851)
Supplement: Supplementary file 1 [file foods-13-03851-s001.zip › foods-3331144-supplementary.pdf]

Lactiplantibacillus plantarum KABP051: stability in fruit juices and production of bioactive compounds during their fermentation

Francesca Rizzi[1,2], Bibiana Juan[1], Jordi Espadaler-Mazo[2], Marta Capellas[1] and Pol Huedo[2,3]*

[1]Centre d'Innovació, Recerca i Transferència en Tecnologia dels Aliments (CIRTTA), XIA, TECNIO, Departament de Ciència Animal i dels Aliments, Facultat de Veterinària, Universitat Autònoma de Barcelona (Cerdanyola del Vallès), 08193 Barcelona, Spain.
[2]R&D Department, AB-Biotics S.A. (Part of Kaneka Corporation), 08174 Barcelona, Spain.
[3]Basic Sciences Department, Universitat Internacional de Catalunya, 08195 Barcelona, Spain.
*** Correspondence:**
Corresponding Author
huedo@ab-biotics.com

Keywords: probiotics, fermentation, functional foods, bioactive compounds, fruit juices

Supplementary Tables

Table S1. Concentration of SCFA (mM) in orange, apple and peach juices detected by targeted metabolomics.

	**Orange**			**Apple**			**Peach**		
**SCFA**	**B**	**P**	**PF**	**B**	**P**	**PF**	**B**	**P**	**PF**
Formic acid	9.78 ± 1.073	6.8 ± 1.67	34.8 ± 17.6	6.84 ± 0.85	9.22 ± 0.15	8.13 ± 1.135	11.9 ± 9.36	8.14 ± 4.18	11.5 ± 4.64
Acetic acid	0.98 ± 0.1	0.81 ± 0.03	1.96 ± 0.11*	2.31 ± 0.08	2.45 ± 0.33	2.55 ± 0.156	2.12 ± 0.19	2.09 ± 0.34	3.51 ± 0.32*
Propanoic acid	<LOD	<LOD	0.03 ± 0.02	0.078	0.1 ± 0	0.06 ± 0.028	0.183	<LOD	0.028
Butanoic acid	0 ± 0.001	0	0.01	0.001	0.001	0.001	0.02	0.02	0.02
Pentanoic acid	0 ± 0.00005	0	0	0.001	<LOD	0 ± 0.0001	0.002	0	0
Hexanoic acid	<LOD	0.01 ± 0	0.04 ± 0.01	0.031	0.03 ± 0.02	0.052	0.02 ± 0.01	0.025	0.03 ± 0.01
Heptanoic acid	0.0118	0.017	0.02 ± 0	0.0061	<LOD	<LOD	0.01	0.017	0.01
2-methyl-propanoic acid	<LOD	<LOD	0.01 ± 0.01	0.03 ± 0.02	0.02	<LOD	<LOD	<LOD	0.01 ± 0.01
3-methyl-butanoic acid	0.05 ± 0.011	0.05 ± 0.02	0.14 ± 0.01	<LOD	0.07	0.049	0.09 ± 0.02	0.1 ± 0.01	0.08 ± 0.02
4-methyl-pentanoic acid	<LOD	0.01 ± 0.01	0.01 ± 0.01	0.1 ± 0.08	0.06 ± 0.01	0.04 ± 0.054	0.02 ± 0.02	0.01	0.09 ± 0.06

B: Blank; P: probiotic (non-fermented); PF: probiotic fermented (24 h)

LOD: Limit Of Detection

* $P<0.05$; t-test PF vs B

Table S2. Additional compounds of interest detected, consumed or produced by *L. plantarum* KABP051, in fruit juices. Values are expressed as log2 of Area Under the Curve (AUC) vs blank (B) juices.

		Orange (Log2 vs B)		Apple (Log2 vs B)		Peach (Log2 vs B)	
**Compounds**	**Level**	**P**	**PF**	**P**	**PF**	**P**	**PF**
Adenosine	1	−0.01	−4.01	−0.40	−2.25	−1.43	−3.91
Lactic acid	1	0.27	4.82	0.12	4.99	0.76	4.82
Malic acid	1	0.01	−5.16	−0.03	−7.63	0.12	−9.28
N-acetyl glutamine	1	0.00	5.84	0.71	2.75	1.33	2.97
3-Phenyllactic acid	2a	1.20	3.44	5.29	7.57	1.65	3.30
Acetylcholine	2a	3.65	4.67	5.80	9.21	4.82	5.80
Adenine	2a	1.31	4.58	0.15	−4.13	0.88	−5.69
Cytidine	2a	−0.06	−2.12	−0.14	−4.09	−0.84	−2.17
Glyoxylic acid	2a	−0.04	−3.33	−0.02	−4.66	−0.14	−3.62
Guanosine	2a	−0.09	−3.58	−0.33	−3.09	−1.04	−3.43
N8-Acetylspermidine	2a	0.42	3.74	2.46	3.88	4.07	6.86
Nicotine amide	2a	−0.50	−5.31	−0.30	−5.59	−2.09	−6.80
Pyruvic acid	2a	0.07	2.29	0.28	0.64	0.99	3.49
Uridine	2a	−0.05	−4.03	−0.18	−8.00	−0.62	−5.74
(−)-β-Pinene	2a	−0.07	6.07	−0.51	−1.63	3.05	8.11
N6-Acetyl-L-lysine	2b	−0.03	0.50	0.94	3.03	0.59	1.61
Acetophenone	2b	0.07	0.06	1.51	1.40	1.10	1.14
trans-3-Indoleacrylic acid	2b	0.06	0.03	0.66	−2.90	0.17	−1.25
Ethyl lactate	3	0.78	4.44	1.04	6.03	1.86	7.14
Diacetin	3	−0.01	2.93	−0.08	6.56	0.39	8.29
Ethyl acetoacetate	3	1.28	5.38	0.73	3.25	2.30	3.99
2-Methyl-5-acetonyl-7-hydroxychromone	3	0.96	4.52	0.98	5.81	2.08	7.03

B: Blank; P: probiotic (non-fermented); PF: probiotic fermented (24 h)

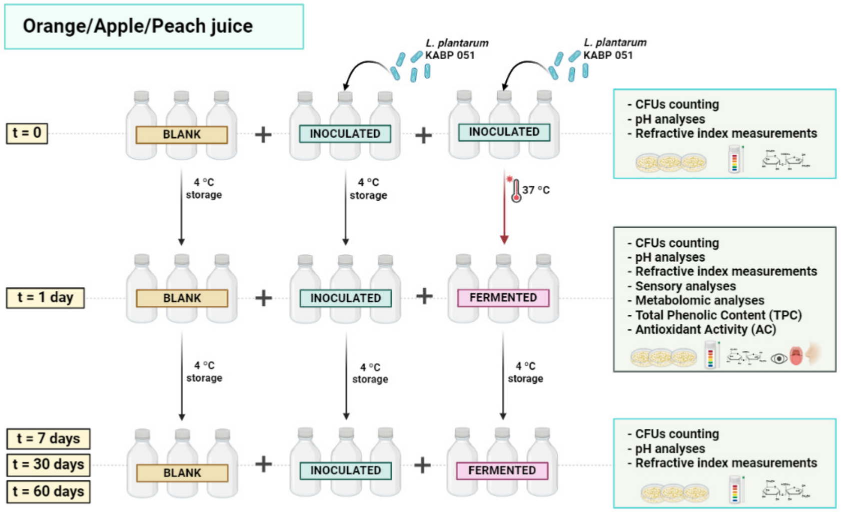

Figure S1. Schematic representation of the experimental design. Three samples for each orange, apple and peach juices were prepared: original fruit juice without probiotic as blank (B), fruit juice with 10^9 CFU/serving of probiotic without fermentation (P), and fruit juice fermented at 37 ºC for 24 h with 10^9 CFU/serving of probiotic (PF). Stability analyses were conducted throughout the shelf life of two months. Metabolomic analyses were conducted on samples obtained at day 1.
